# Void-Induced Ductile Fracture of Metals: Experimental Observations

**DOI:** 10.3390/ma15186473

**Published:** 2022-09-18

**Authors:** Wiktor Wciślik, Sebastian Lipiec

**Affiliations:** 1 Faculty of Civil Engineering and Architecture, Kielce University of Technology, 25-314 Kielce, Poland; 2 Faculty of Mechatronics and Mechanical Engineering, Kielce University of Technology, 25-314 Kielce, Poland

**Keywords:** ductile fracture, material testing, void nucleation, growth, coalescence, microstructure, material characterisation

## Abstract

The paper presents a literature review on the development of microvoids in metals, leading to ductile fracture associated with plastic deformation, without taking into account the cleavage mechanism. Particular emphasis was placed on the results of observations and experimental studies of the characteristics of the phenomenon itself, without in-depth analysis in the field of widely used FEM modelling. The mechanism of void development as a fracture mechanism is presented. Observations of the nucleation of voids in metals from the turn of the 1950s and 1960s to the present day were described. The nucleation mechanisms related to the defects of the crystal lattice as well as those resulting from the presence of second-phase particles were characterised. Observations of the growth and coalescence of voids were presented, along with the basic models of both phenomena. The modern research methods used to analyse changes in the microstructure of the material during plastic deformation are discussed. In summary, it was indicated that understanding the microstructural phenomena occurring in deformed material enables the engineering of the modelling of plastic fracture in metals.

## 1. Introduction

Ductile fracture criteria can be classified based on the physical sense of the quantities defining them. If the criterion includes values derived by macroscopic analysis based on mechanics of deformable bodies, without precisely defined areas of fracture initiation, a global criterion is formulated. The most known and commonly used global ductile fracture criteria (also known as phenomenological) were determined on the basis of such quantities as J integral and crack tip opening displacement δT.

Another approach involves analysis of the stress and strain (or other parameter) fields in the most stressed areas of the material (process zone). The stress and strain state in the process zone determines the strength of the whole element. Such an approach and arising criteria are local. The criterion values of local models depend only on the material properties.

An undoubted advantage of using a local approach to the analysis of the fracture is the independence of the obtained results on the geometry of the samples used. The local approach is based on actual physical phenomena (degradation of the microstructure) that occur in the material subjected to load. In general, understanding and describing these phenomena allow for their inclusion in the cracking description of any element type, and thus the development of a universal, comprehensive method of predicting the durability of structural elements and assessing the safety of their work.

Conducting an analysis according to a local approach requires the use of hybrid methods: obtaining data from experimental research (with accompanying in situ analyses, e.g., video extensometer, recording of acoustic emission signals, using computed tomography techniques) and supplementing them with numerical, metallographic and fractographic analyses.

Among the physical phenomena taken into account in the local approach to fracture, the nucleation and growth of microvoids in the structure of the material subjected to plastic deformation is of particular practical importance. Observation of this phenomenon is the basis for formulating theoretical models of nucleation, growth and coalescence of voids, which in turn enables the establishment of models of plastic materials with microdamages. These issues are therefore crucial for assessing the safety of structural elements. This article is a literature review on the microstructure observations and void growth in deformed metals. Particular emphasis was placed on ductile fracture associated with substantial plastic deformation, without taking into account cleavage cracking. The results of pioneering works from the second half of the twentieth century, as well as the latest results obtained with advanced research tools (such as microtomography), are discussed. The structure of the article is divided into sections according to the different mechanisms in ductile fracture: Section 2 provides general information on void-induced failure; further, the nucleation of voids (Section 3) and subsequent stages of their growth and coalescence (Section 4) are described.

## 2. Cracking of Metals by the Development of Voids

**Figure 1:**
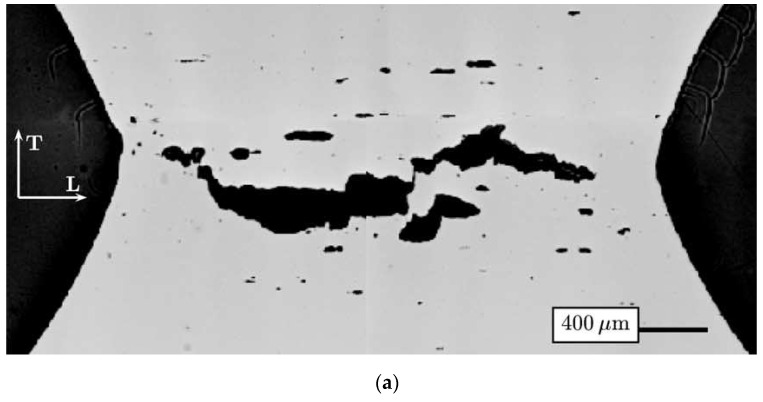

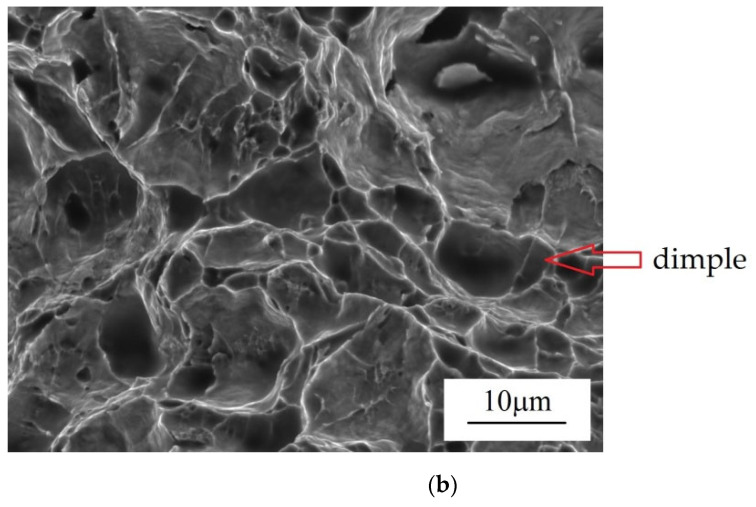
Void-induced failure of metals: (**a**) Formation of a macroscopic crack due to void growth and coalescence in X52 steel [1]; (**b**) Fracture surface of a tensile S355 steel specimen and dimple microstructure resulting from void development (authors’ own study).

Void initiators are primarily defects in the crystalline structure (point, linear and planar defects) and second-phase particles, which can be introduced into the material microstructure intentionally (e.g., metal matrix composites) or constitute an undesirable contamination of the material. All these discontinuities, being local stress concentrators, under plastic deformation create voids with sizes in the order of tenths of a micrometre. As the plastic strain increases, the voids created in this way increase their size many times and then coalesce, forming a larger defect and leading to failure. An example of a sample structure in which a fracture developed as a result of the growth and coalescence of voids is presented in Figure 1a.

Figure 1b presents a microscopic photograph of the fracture surface of the S355 steel sample damaged due to the development of voids. The characteristic dimple microstructure is observed, in which the voids of a few μm in size are surrounded by plastically deformed ligaments (light bands in Figure 1b) [2].

**Figure 2:**
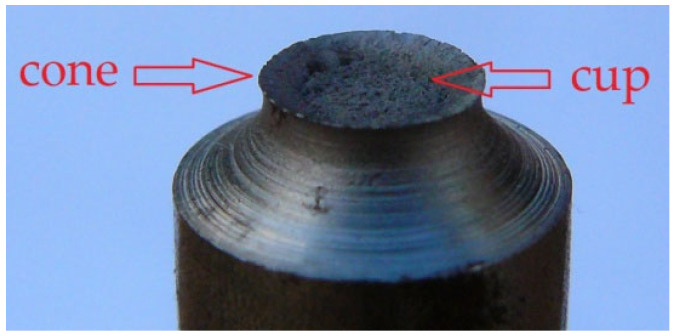
An example of a S355 steel specimen failure due to void development. Characteristic cup–cone shape is clearly visible [authors’ own study].

A separate issue is the macroscopic observation of fracture surfaces at void-induced failure. Ductile fracture of a round tensile bar has been relatively well-documented in the literature [3]. Initially, voids develop mainly in the centre of the sample, whereby the crack propagates approximately perpendicular to the sample axis, in the plane of the minimum cross section (in the neck area). As the crack approaches the sample surface, the influence of constraints decreases, so the crack deviates from its initial plane. As a result, the fracture of the sample takes the form of a cup–cone fracture (Figure 2), in which the central part is flat (cup) and an inclined area (cone) is formed around it.

An in-depth analysis of microstructures with the use of different methods allowed for the development of the systematics of the mechanisms of the formation of voids. This applies to both the nucleation of the voids and their subsequent development.

The initial experimental observations of the development of voids in plastically deformed metals took place at the turn of the 1950s and 1960s [4]. Since that time, a number of research methods have been developed to observe the expansion of voids in metals. The most common studies include the use of optical microscopy and scanning electron microscopy (SEM). The first attempts of 3D characterisation of voids involved the use of indirect methods, such as change in elastic stiffness [5,6] and change in resistivity and density of the sample [7,8]. A common disadvantage of indirect methods is the ability to measure only the volume fraction of voids, without taking into account their distribution, shape, size and other geometric features [9]. The use of X-ray tomography offers much more possibilities in this respect.

Section 3 describes the results obtained with the above-mentioned methods, taking into account different nucleation mechanisms and different types of void initiators. Section 4 presents the observation of the growth and coalescence of voids, as well as characterises the basic analytical models of these phenomena.

## 3. Void Nucleation in Metals

### 3.1. Overview

In general, voids are nucleated in the vicinity of stress concentrators on a microscopic scale [10]. In the literature, the most frequently mentioned mechanisms include the formation of voids at the intersection of the slip bands [11], at the grain boundaries [12], at twin boundaries and at vacancy clusters [13], but most importantly around inclusions and precipitates [14,15,16].

### 3.2. Nucleation with No Particles Involved

In the absence of material discontinuities, the formation of voids is observed as planar slip band decohesion or grain boundary decohesion (homogeneous nucleation). One of the first widely known studies in which the participation of dislocations in the formation of voids was experimentally confirmed was developed by Gardner et al. [12], who noticed that the dislocation structures in iron and beryllium crystals evolved into cells at high strains. The boundaries between individual cells were characterised by sufficient surface energy to create a void, even in the absence of other internal stress concentrators.

The observations of the development of voids in the Nb-Cr-Ti alloy [11] showed that the voids formed in this way are characterised by a flattened shape.

**Figure 3:**
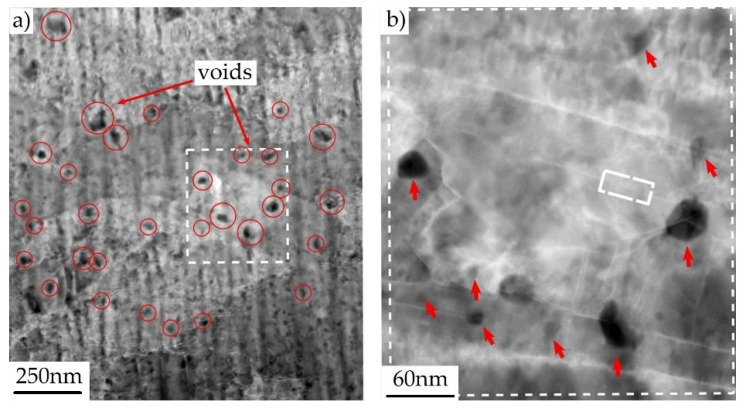
(**a**) HAADF-STEM image presenting nanoscale voids (black areas) in copper subjected to tension (plastic strain); (**b**) Enlargement of the boxed area in (**a**), from [20]. White rectangle in (**b**) indicates the twin boundary.

The concept of void nucleation as a result of vacancy condensation and the presence of dislocation boundaries has been known for a long time [17,18], but only modern research techniques allowed for a better understanding and documentation of this phenomenon [19]. For example, in [20], by investigating the fracture mechanism of copper containing copper oxide particles, it was found that the voids are first nucleated at the nanoscale, most often without any relation to the presence of the second-phase particles. An example of a photograph of nanovoids, made with the use of the high-angle annular dark-field scanning transmission electron microscopy (HAADF-STEM), is shown in Figure 3.

In the next stage, only a small quantity of the nanovoids grew to the microscale, contributing to the initiation of cracking. It was noted that all microscale voids were associated with a dislocation boundary. By carefully analysing the microstructure of the material in the neck area, the authors distinguished three basic groups of microvoids, depending on their location and the mechanism of formation, namely: (i) voids related to intragranular, inclusion-free dislocation boundaries; (ii) voids associated with the inclusion-free intersection between one or more dislocation boundaries and one or more grain boundaries; (iii) voids associated with an inclusion intersected by a dislocation boundary.

As mentioned before, voids can be initiated by grain separation. This mechanism is favoured by the following conditions: high share of the hydrostatic stress; small value of the void spacing to void diameter ratio; and high value of the precipitate-free zone (PFZ) thickness to void spacing ratio [21]. 

### 3.3. The Role of Second-Phase Particles

**Figure 4:**
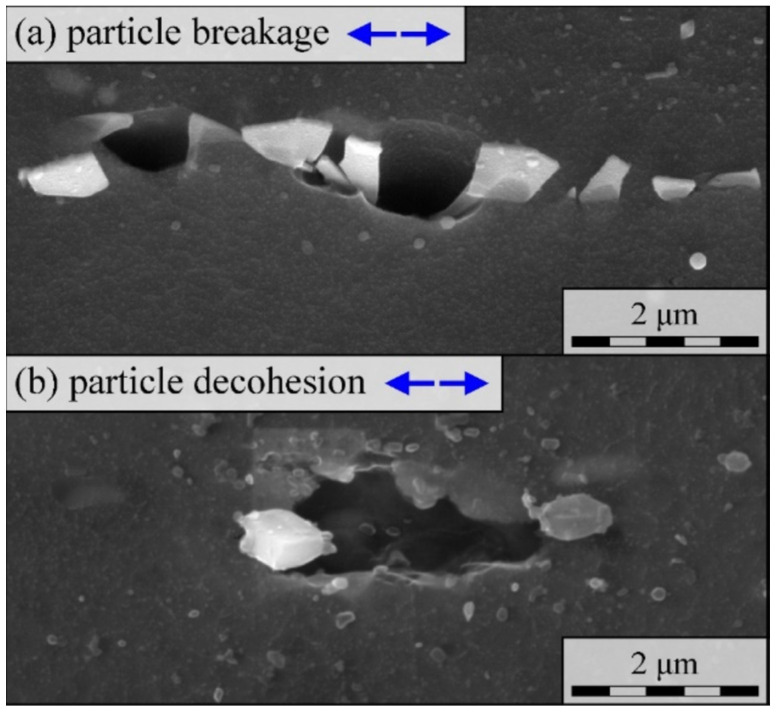
Two mechanisms of void nucleation in AZ31 magnesium alloy: (**a**) Particle fragmentation/fracture; (**b**) Matrix–particle separation [23].

In technical alloys, voids are often initiated by the failure of second-phase particles, randomly distributed over the material matrix (heterogeneous nucleation). The presence of a hard particle locally limits elastic and plastic deformation of the matrix, which in turn causes local stress concentration. With the increase in plastic strain, the stress value also increases, which ultimately leads to the particle cracking or its separation from the matrix [22]. As the result, a void is formed (Figure 4).

The occurrence of one of the two mentioned mechanisms depends largely on the mechanical properties of the matrix and the particle (strength, ductility) and the strength of the matrix–particle interface. In general, the phenomenon of particle separation is primarily observed in relatively soft, ductile matrices. However, high yield stress and hardening exponent of the matrix as well as the high particle stiffness promote particle cracking. 

Moreover, the nucleation mechanism depends on the particle geometry (size, shape) and its orientation with respect to the direction of the principle tensile stresses. Larger particles usually break, as do elongated particles parallel to the principal stress direction. In practice, engineering materials have different void populations; therefore, both mechanisms occur simultaneously. The local stress state is also of great importance, namely the predominance of normal stress over shear stress, which most often forces the particle fracture [24,25].

Due to the heterogeneous stress state changing with time, the nucleation of voids is continuous across the entire range of plastic strain. In other words, the development of microdamage includes the simultaneous growth and coalescence of existing voids as well as the nucleation of new ones [26].

Among the experimental studies, the process of nucleation of voids and microcrack formation by fracture of silicon particles in Al-Si alloys is relatively well-documented. The most important results described in the literature are summarised in [27]. As shown in [28,29], cracking of silicon particles and their separation from the matrix were observed already at strain values of 1–2%, while the quantity of damaged particles increased linearly with the increasing strain [30]. According to the results of observations described in [31], a maximum of 10% of the particles were damaged in the samples subjected to tension or compression. In general, larger particles crack first [32], which is often explained by a greater probability of internal defects in this type of particles. Smaller-sized particles tend to detach from the matrix, initiating voids.

As noted in [30], the phenomenon of fracture of silicon particles in the Al-7Si-0.4Mg alloy subjected to tension and bending occurs mainly in the case of elongated particles. The authors also analysed the structure of the alloy deformed to failure. In the areas with a homogeneous deformation (a few mm from the fracture surface) between 3 and 10% of the Si particles cracked. However, in the immediate vicinity of the fracture surface, the proportion of fractured particles locally increased to about 15–20%. In the coarser structures, the fracture of the particles was sudden and occurred at low strains. The development of microdamage in the finer structures was gradual.

### 3.4. Estimating Particle Strength

To understand the phenomenon of void nucleation by fracture of the second-phase particles, it is important to estimate their mechanical properties, especially strength. The first and best-known attempts were described in [33,34]. In [34], the microscopic maps of the locations of cracked and separated MnS inclusions were compared with the numerically determined stress and strain distributions, which gave rise to assessment of the critical fracture stress of MnS particles of about 1120 MPa. Additionally, in a similar manner, the critical stress of separation of the MnS particle and the matrix was determined, where the stress value was about 810 MPa.

A similar approach was used in [35], but this time the X-ray technique was used to assess the particle deformation. The tests were carried out on a sample made of an aluminium alloy, subjected to pure bending. Using the X-ray method, the strain values of silicon particles were determined, and then the stresses in the particles were determined as a function of the measured strains.

The typical research methods used so far did not take into account the internal defects of the particles; therefore, the obtained results are not precise.

**Figure 5:**
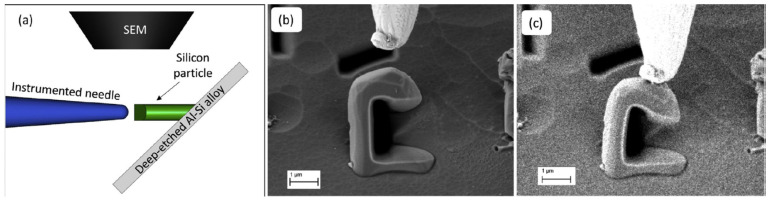
Strength test of silicon microparticles in A356 aluminium alloy: (**a**) Scheme of the micromechanical test; (**b**) C-shaped particle and the tungsten tip before test; (**c**) Last frame before particle fracture, from [36].

The significant progress made in recent years in the field of methods of microstructural materials testing allowed for a more in-depth analysis of this issue. An interesting attempt to determine the strength of silicon particles in the A356 aluminium alloy is described in [36]. In order to expose the silicon particles, the ground surface of the sample was subjected to deep etching with a mixture of phosphoric, acetic and nitric acids. As a result, the particles to be tested were exposed to a height of several dozen micrometres. In the next step, using the focused ion beam (FIB) method, the geometric notch of the selected particles was cut. The prepared particles were subjected to an eccentric compression test in which the load was transferred to the particle by a tungsten needle. The test scheme is shown in Figure 5. The entire course of the process, up to the particle fracture, was recorded using a scanning electron microscope (SEM) with the video recording. In the next stage, based on the values of the measured force, the evaluation of the stress values in the particle was performed, using the simplified analytical method and with the use of the finite element method (FEM). For the defect-free particles, the particle strength was determined to be around 16 GPa. Detailed visual inspection of particles using SEM, combined with FEM modelling, made it possible to evaluate the effect of different types of particle defects on their strength. It was found that the presence of defects can reduce the strength of the silicon particles to 2–3 GPa.

**Figure 6:**
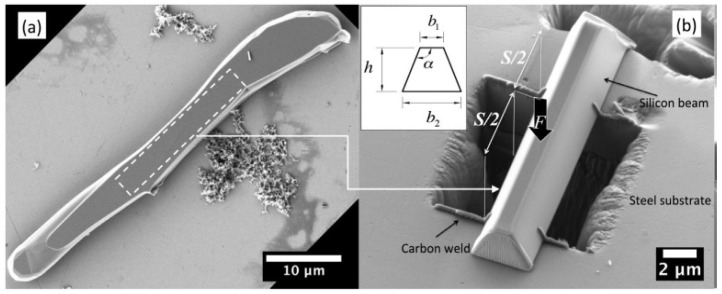
(**a**) Eutectic silicon particle extracted from the Al-Si alloy; (**b**) Microscopic three-point bending specimen prepared from the particle in (**a**), from [37].

The article [37] also provides an example of particle strength assessment by means of a microscopic three-point bending test. First, the silicon particles were extracted from the Al-Si alloy by dissolving the aluminium matrix. Then, after cleaning and selecting the particles, microscopic beams were cut from them using the above-mentioned FIB technique. The beam prepared in this way was placed on a steel base with a cut-out hole and subjected to load. Figure 6 illustrates the tested sample. The results obtained during the experiment were compared with the results of analytical and numerical calculations, which resulted in determining the strength of silicon at the level of about 9 GPa, assuming no particle defects.

A significant technical problem during the microscale strength tests is the measurement of deformations and stresses. Recently, the Raman spectroscopy technique offers a wide range of analysis of stress values on a microscopic scale, and thus also the evaluation of particle strength. The Raman effect is related to inelastic light scattering. After filtering, the light scattered on the sample goes to the spectrometer, which records its spectrum. The spectrum is presented as a function of the Raman shift, defined as the difference between the frequency of the scattered light and the input light [38]. In materials science, spectrum analysis enables the recording of the stress values, especially in the case of uniaxial stress states. However, the authors of [39] present the methodology of plane stress analysis in Si wafers on a microscopic scale.

A more complex analysis is discussed in [40], where the stress state in eutectic silicon particles in the Al-Si alloy was analysed. The alloy was tested in as-received condition under uniaxial loading. Cracking of silicon particles was observed already at the stress value of 600 MPa. Importantly, the use of Raman spectroscopy enables the assessment of the effect of particle size and its neighbourhood on the strength.

As already mentioned above, the second mechanism of void formation involves the decohesion of the matrix and the second-phase particles. Due to technical difficulties in a detailed, experimental study of this phenomenon, there are relatively few works of this type. Thus, numerical analyses are of particular importance. The solutions described in the literature are most often theoretical in nature. For example, in [41,42,43] a hypothetical aluminium matrix–silicon particle interface strength was determined in the range of about 4–7 GPa in tension, while the shear strength was only about 300 MPa. As this paper deals primarily with experimental research, these issues will not be described in detail here. More information on the simulation of this phenomenon, and the use of cohesive models, can be found in [44,45,46,47].

### 3.5. Effect of Martensite Cracking in DP Steels

As it has been shown in many studies, for example [48], the mechanism of void formation in dual-phase (DP) steels is only slightly based on the fracture and separation of the second-phase particles, because the fracture of martensite plays the most important role. The development of microdamage of DP1000 steel subjected to tension was analysed in detail in [48]. Strength tests were carried out inside the scanning electron microscope (SEM) chamber and stopped at regular intervals, each time photographing the microstructure of the material in the selected area. Cracking of the particles was observed at overall small strain, of the order of 2%. With the increase in plastic deformation, the voids created in this way grew, but no crack development in its vicinity was observed. As the strain value was increased further, the martensite phase cracked. Due to increasing deformation, the crack turned into a void, which, being a stress concentrator, became the cause of crack propagation in the ferrite. The high intensity of this phenomenon accounted for its dominant role in the failure of DP1000 steel.

Using advanced research methods, the authors of [48] attempted to estimate the local values of strains and stresses accompanying martensite cracking and void initiation. The obtained photographs of the microstructure were processed using the digital image correlation (DIC) technique. The photograph of the undeformed structure was divided into subset windows, then an algorithm was applied to track these areas in the photographs of the deformed structure. The results obtained in this way made it possible to determine the vectors of displacements and local deformations. Further, the experimentally determined values of displacements were used as boundary conditions in the finite element method (FEM) model of the tested sample, which allowed for the estimation of martensite cracking stress at the level of about 1700 MPa.

The dominant role of martensite cracking in the formation of voids in DP steels, mainly of the coarse structure, was also emphasised in [49]. This is the leading mechanism at low strains. At a later stage, voids were formed mainly by the decohesion of ferrite and martensite. The latter mechanism dominated in steels with the finer structure in the entire range of deformation. The occurrence of the decohesion is mainly attributed to the lower deformability of martensite.

Observations of martensite cracking in DP600 steel under uniaxial tension at low strain values were also described in [50], although as the authors point out, the importance of this mechanism in the entire process of void nucleation is not great. The process of fracture and separation of second-phase particles led to the formation of a few voids, which, however, were characterised by large dimensions, and therefore their area fraction was significant.

The dominant mechanism for the formation of voids was, as in [51], decohesion at the interface between ferrite and martensite, observed in the entire range of deformation of the tensile sample. The voids were initiated mainly at the interfaces perpendicular to tensile stresses and enlarged along the ferrite grains. The authors of [50] also noticed that with the increase in strain, the mean size of the voids decreased, which indicates a high intensity of nucleation of new voids also immediately before failure.

The authors of [52] drew similar conclusions. While examining the development of microdamage in DP600 and DP800 steels under uniaxial tension, it was noticed that at low strain, the void initiators were the globular aluminium oxide inclusions, but with higher deformations the voids were formed near to the ferrite–martensite interfaces as well as in the ferrite matrix and close to martensite islands.

### 3.6. Quantitative Description of Void Nucleation 

Regardless of the single void nucleation analysis, it is important to evaluate the void nucleation globally, determining the number of nucleated voids as a function of remote strain and the location of the analysed area. In this case, it is particularly important to continuously track changes in the microstructure of the material throughout the deformation range up to the failure.

In recent years, the widespread use of the X-ray microtomography method gave great opportunities in this regard, and has contributed to a much better understanding of the phenomenon of failure and void development [9,53].

For example, in [54], the tomography method was used to assess changes in the microstructure of JIS SUM24L free-cutting steel under uniaxial tension. The tests were carried out on tensile specimens subjected to uniaxial stress state. The tests were interrupted at various stages, each time taking tomographic photographs of the microstructure in one selected area. In order to precisely determine the value of strain, especially after necking, changes in the width of the specimen were recorded using tomographic images. As part of the microtomographic research, in the first step, the region of interest (ROI) was distinguished, along with the voids present in the unstrained material. The photographs were then binarised, indicating base material and voids. In the next stage, a 3D labelling algorithm was applied to the binarised images, and then the volume and position of each of the detected voids was determined. In this way, over four thousand voids and the second-phase particles were marked in the ROI, which allowed for their tracking in the entire range of given deformation.

A separate issue was the development of an algorithm that allows for tracking each of the voids in subsequent stages, with increasing values of strain. The adopted procedure included the determination of a transformation matrix that was calculated by minimising the sum of distance difference between corresponding pairs of objects detected in subsequent stages of loading. Due to the heterogeneity of deformation and the different quality of individual photos, the obtained results were not fully accurate, hence the matching probability parameter $M_{p}$ was introduced into the analysis. The algorithm developed in this way took into account the translation and rotation of the voids related to the occurrence of plastic deformation. A detailed description of the voids tracking algorithm is presented in [55,56].

**Figure 7:**
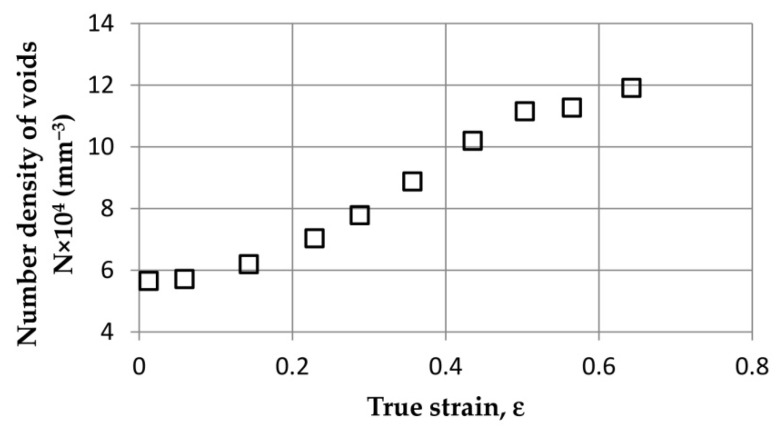
Experimentally determined relation between density of voids and plastic strain in JIS SUM24L free-cutting steel, from [54].

The results of the observation of microstructure changes indicate that the nucleation of voids was continuous throughout the analysed range of deformations, up to failure. This phenomenon is well-illustrated by the graph in Figure 7, where the number of voids in the unit volume [mm^3^] of ROI was determined as a function of true strain. From the very beginning, a constant increase in the number of voids is visible. Just before failure, one can see a flattening of the curve, which seemingly means a reduction in the intensity of void nucleation at this stage. In fact, as noted by the authors of [54], nucleation of voids is still present; however, at this stage, the ductile fracture process is controlled primarily by the void coalescence phenomenon, which explains the flattening of the curve in Figure 7.

**Figure 8:**
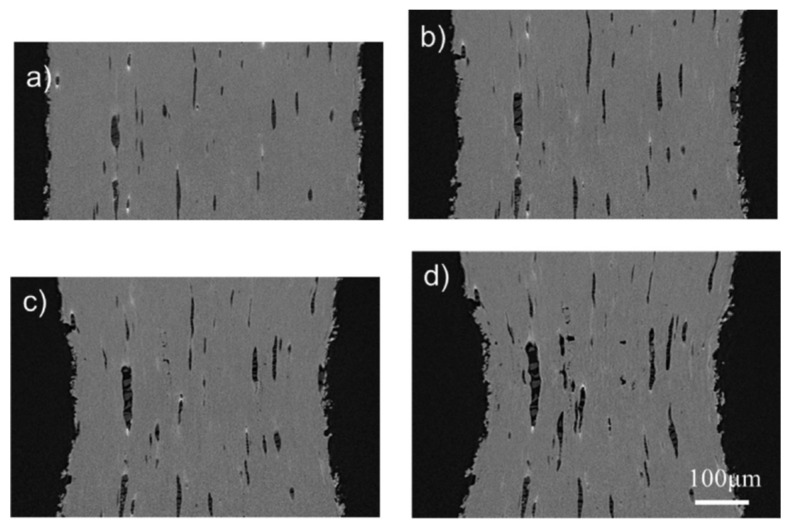
Free-cutting steel (JIS SUM24L grade) microstructure at different strains: (**a**) 0; (**b**) 0.23; (**c**) 0.50; (**d**) 0.64, from [54].

Figure 8 presents tomographic cross sections of ROI, made at different strains. The vertical axis in the individual figures is equated with the direction of loading. Dark areas represent voids, while lighter areas represent the matrix. In Figure 8b (with the strain 0.23), the neck is clearly visible.

Detailed analysis of the individual pictures shows that the nucleation of voids occurs primarily at the interface between the matrix and the particles. The voids are also initiated at the places where the particles of the second phase break. As can be seen from the comparison of the subsequent photographs, some voids seem to disappear as the strain increases. This is due to their rotation and displacement, because the strains are not uniform throughout the tested sample. The void-tracking algorithm described above allowed for the inclusion of this phenomenon in the void development analysis.

Additionally, on the basis of the obtained results of tomographic examinations, changes in the volume and diameter of voids as a function of plastic strain were determined. While, as predicted, the total volume of the voids in the sample increased with increasing strain, the mean diameter of the voids was almost constant, regardless of the strain level. The authors of [54] indicate the nucleation of new small voids at higher strain levels as a possible reason.

The authors of [57] also emphasised the key role of small voids in the initiation of fracture. Using the 4D X-ray microtomography technique (3D + time), changes in the microstructure of SA508 steel were analysed in the entire range of tensile strains up to failure. It was observed that large Al_2_O_3_ and MnS_2_ particles (with sizes ranging from several to tens of micrometres) cracked or separated from the matrix at zero values of plastic strain (elastic range). Considering the fact that such particles were scattered and spaced far apart, the voids they initiated did not coalesce, and therefore their contribution to the fracture initiation was insignificant. On the other hand, the elongation of voids along the tensile axis was observed, but the their contraction in the perpendicular direction was not significant. Moreover, the rotation of the elongated voids took place as a result of the increase in the value of shear stresses after the formation of the neck.

**Figure 9:**
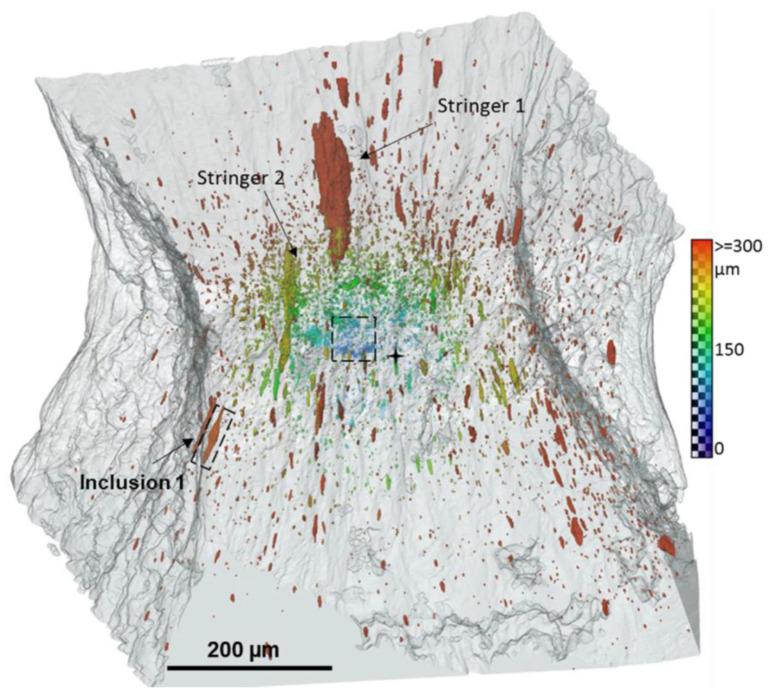
Microtomographic 3D image of the distribution of voids in SA508 steel, at the onset of failure, in the central part a cluster of small voids initiating a macroscopic defect is present, from [57]. The colour scale indicates the distance between void and sample centre.

On the contrary, small particles of cementite (with a size of the order of 100–500 nm) detached from the matrix when the remote strain of the order of two was achieved. The initiation of small voids was, however, sudden. A large accumulation of small voids favoured their coalescence, which led to formation of a crack. The microtomographic image of the microstructure of the sample before failure is shown in Figure 9. In the central part of the region of interest, a cluster of small voids initiating a crack is clearly visible.

Larger elongated voids predominate at a greater distance from the centre of the sample. The two largest voids (marked in the figure as stringer 1 and 2) were formed from an agglomerate of particles. Additionally, the authors of the paper carefully analysed the void nucleation around the inclusion marked as “inclusion 1” in Figure 9, indicating the mechanism of matrix–particle decohesion.

### 3.7. Effect of Stress State on Void Nucleation Intensity

**Figure 10:**
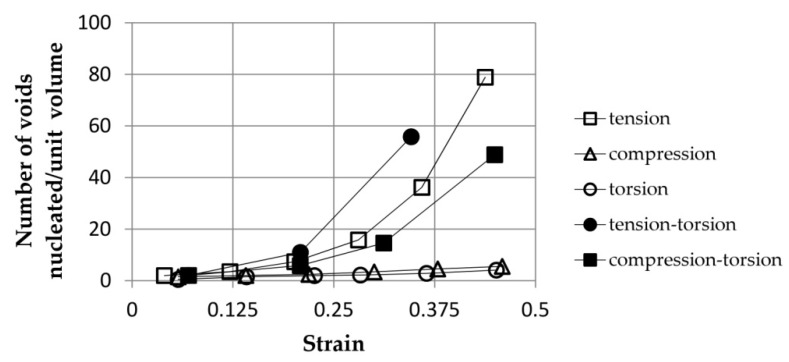
Effect of the loading type on the intensity of void nucleation in cast Al-Si-Mg alloy as a function of strain, based on [10].

In the previously mentioned work [10], the influence of the type of load on the intensity of void nucleation was determined. Figure 10 illustrates an exemplary dependence of the number of nucleated voids per unit volume of the cast Al-Si-Mg sample as a function of strain for various loading conditions. The results were obtained using the proprietary analytical void nucleation model. The lowest intensity of void nucleation was obtained for compression and torsion. As expected, among the simple load cases, the voids in the sample subjected to tension showed the highest nucleation intensity. The simultaneous action of tension and torsion resulted in the most intense nucleation of the voids.

Regardless of the above, the authors of [10] defined the material constants of void nucleation for simple loading conditions (tension/compression, torsion).

Recently, the authors of [58] conducted a thorough experimental analysis of the impact of the stress state on void nucleation in DP780 and CP800 steels. In order to obtain different components of the stress state, various strength tests were carried out: simple shear, hole tension, v-bending and biaxial tension. In each case, the tests were stopped, recording the material microstructure in the region of interest, using microtomography. Nucleation intensity was measured as the average number of nucleated voids in 1 mm^3^ of material in the process zone. At failure, the highest number of voids (about 30,000/mm^3^) was observed in the biaxial tension specimens. In the case of hole tension, this value was much lower and ranged from about 9500 to 18,000, depending on the material tested. The specimens subjected to shearing were characterised by the lowest nucleation intensity, i.e., at failure, values between 3000 and 4000 voids in 1 mm^3^ of material were recorded.

One of the most frequently analysed issues related to the dependence of void nucleation on stress state is the influence of stress triaxiality T on the value of the strain needed to initiate the void. Stress triaxiality T describes the effect of the spherical component of the stress tensor (hydrostatic tension or compression) and is defined as the quotient of the mean stress (arithmetic mean of the principal stresses) and the Huber von Mises stress. The works published so far, for example, [59,60], unanimously indicate that the increase in triaxiality (increase in the hydrostatic pressure share) is accompanied by an exponential decrease in the value of nucleation strain.

The author of [61] drew similar conclusions, at the same time indicating the large influence of the Lode parameter on nucleation and the growth of voids. The Lode parameter takes into account the influence of the third stress tensor invariant. According to Yu [61], the value of the Lode parameter does not significantly affect the value of the void nucleation strain; however, it plays an important role, as interfacial cracks nucleate from different positions for different Lode parameters and propagate in different patterns. This is due to the fact that the Lode parameter changes the principal stress distribution, even at constant triaxiality.

Han et al. [62], studying the development of voids in QP980 steel under shear load, noticed that a large number of small voids (less than 5 μm in size) was formed at phase interfaces. In turn, a few microvoids generated from inclusions had more than 5 μm.

The phenomenon of the development of voids under shear was also analysed in detail by the authors of [63], also indicating the low intensity of nucleation in these conditions. The combination of microtomographic tests with FEM simulation allowed for the determination of the mechanism of ductile fracture of FB600 steel, initiated by separation of the matrix from CaO particles. The voids created in this way grew towards the largest local deformations, forming microcrack-like defects. As noted, a shear-band type of failure was formed on the microscopic scale even with a small volume fraction of voids, of the order of 0.015%. The void volume fraction measured before failure did not exceed 0.1%.

In recent decades, computer simulations have made a huge contribution to understanding the phenomena of void development [64]. As this article focuses primarily on experimental observations, the review of FEM results will not be discussed in detail here. However, it is worth paying attention to molecular dynamics simulation [65,66], which offers new possibilities compared to traditional continuum solutions, as it enables material modelling at the atomic level. For example, in [67], the mechanism of decohesion of the AlCu_2_ particle and the aluminium matrix was analysed. In the first stage, the breaking of the bonds between single-inclusion and matrix atoms was observed, which initiated the particle separation. In the next stage, the crack grew steadily, with no dislocation involved. The fracture development in this case was driven by the lattice trapping phenomenon. After the fracture reached a critical size, nucleation of Shockley partial dislocations at the crack tip was observed. Then, the dislocations moved from the particle towards the matrix, whereby the rate of crack propagation increased suddenly, leading to the complete separation of the particle and the matrix. The authors called this stage of separation *dislocation-mediated delamination*.

## 4. Void Growth and Coalescence in Metals

### 4.1. Mechanisms of Growth and Coalescence of Voids

In the course of the realisation of ductile failure, after nucleation (characterised in the previous section of the article) due to plastic strain and hydrostatic stresses, the voids in the material increase [68,69]. With the action of strain, the voids grow, change their shape and move, changing their position. With stable void growth, plastic strain forms relatively uniformly in the material. However, from a certain point, the strain localises between adjacent voids. Outside the plane of strain localisation, the material undergoes elastic unloading. The occurrence of local strain localisation limits the ductility of materials. Two mechanisms for the realisation of strain localisation have been identified. The first involves the softening of strain through factors such as microstructural changes, thermal interactions and damage evolution. There is a local degradation of the load-carrying capacity of the material, resulting in strain localisation in a thin band [70,71,72,73]. The initiation of strain localisation depends on a number of factors, including stress state, material properties and material porosity [74,75,76].

**Figure 11:**
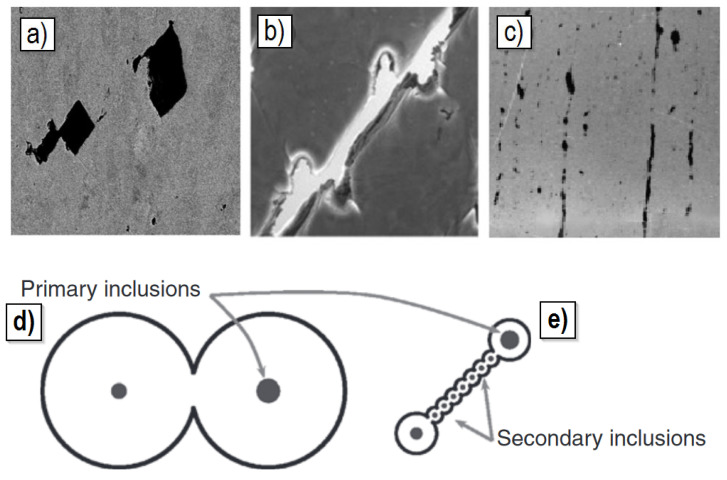
Methods of realising void coalescence in materials (example in steel): (**a**,**d**) Internal necking; (**b**,**e**) Shear coalescence; (**c**) Necklace coalescence [24,78,79].

The second mechanism of strain localisation is associated with the phenomenon of void coalescence. There is a local instability, conditioned by the interaction between adjacent voids in the material. The moment of localisation of plastic strain is identified with the beginning of the process of void coalescence. Once the coalescence process has started, the kinematics of void enlargement differ significantly from the kinematics of void growth prior to this mode of instability. For void coalescence induced by macroscopic strain localisation, the width of the localisation band is narrower. This is due to the restriction of deformation to areas (ligaments) between adjacent voids [76]. The process of void coalescence becomes the direct cause of the initiation and growth of a ductile crack in the material. There are three ways to realise void coalescence [77]:

(1) Internal necking (Figure 11a,d);
(2) Shear coalescence (Figure 11b,e);
(3) Necklace coalescence (Figure 11c).

The internal necking mechanism is the most commonly observed way of the material void coalescence process, initially observed by Argon [33]. With the internal necking process, the plane of localisation is almost perpendicular to the main direction of the applied load. During the process of coalescence of voids, a reduction in the area of the intervacancy ligament can be observed, which is similar to the necking phenomenon during the tensile test of the specimen under uniaxial loading [77,80,81,82,83]. A large contribution to understanding the process of coalescence of voids according to the internal necking mechanism was made by micromechanical analyses involving numerical calculations using the finite element method [71,84,85,86,87]. 

The second distinguished method of coalescence is the mechanism involving localised shear occurring between initial voids (of large size), when the voids are distributed along a line inclined at 45° to the main direction of loading (Figure 11b). The stress state accompanying the development of the failure process according to the void mechanism is the subject of many scientific studies. The stress state is most often defined by the stress triaxiality factor. However, the value cannot unambiguously describe the effect of stress triaxiality on void growth and coalescence. For a given value of the triaxiality factor, more than one stress state exists. The necessary information is very often extended by the magnitude of the Lode parameter. The development of the stress state in ductile fracture was the subject of the work. These quantities were determined on the basis of experimental results and numerical calculations. Selected results will be cited later in this work (Section 4.3). Coalescence in this case can occur according to the so-called “void sheeting” mechanism [88]. Plasticity is localised in shear bands containing small secondary voids; large primary voids are connected through coalescence of these secondary voids. This coalescence mechanism is often observed in high-strength materials with low-to-medium strain-hardening capacity [77,89]. 

A third possible mechanism for the coalescence of voids is called necklace coalescence (Figure 11c). It is the least commonly occurring in materials. The mechanism involves localisation in a direction parallel to the action of the applied load. This mechanism was observed in areas of voids, which are distributed in elongated concentrations. The mechanism of necklace coalescence is considered to be of major importance in the process of occurrence of ductile delamination cracking [77,90,91].

Many elements influence the development of the growth process and the coalescence of voids in ductile failure. These may include the state of stress in the material (which can be defined by the stress triaxiality factor, the Lode parameter), the contribution of shear stress and the level of plastic strain, taking into account the level of critical strain [92]. On the basis of the above quantities and a wide range of experimental and fractographic studies, numerical calculations, various failure models have been proposed, a selection of which will be discussed later in this paper. 

### 4.2. Classical Models for the Growth and Coalescence of Voids

Of the classic local models describing the process of void growth, mention should be made of the solution proposed by McClintock [93]. In his work, he analysed the growth of a cylindrical void in a rigid plastic material under axisymmetric loading, assuming a plane strain condition. The model assumes that the relative volume of voids reaching a critical value will result in crack initiation. As a continuation of McClintock’s study, an approximate solution for spherical void growth was proposed by Rice and Tracey [94]. The void growth model developed by Rice and Tracey allowed for the formulation of a fracture criterion, specifying that a crack will be initiated if the normalised void radius reaches a critical size. Similar results were obtained in [34,95,96,97]. In the cited papers, it was shown that the level of plastic strain and stress triaxiality have a significant impact on the realisation of the void growth process. The cited works formed the basis for further research and development of further local ductile fracture models. 

A well-known model describing the phenomenon of void growth in ductile fracture is that by Gurson [98]. The author, with a similar methodology to the work of Rice and Tracey [94], developed an approach to analyse plastic flow in porous material assuming material continuity. Gurson included in the model the interaction between voids and the effect of void growth on material softening. A modification of Gurson’s model was proposed by Tvergaard and Needleman [3]. The authors made a change in the definition of the relative volume of voids in the material and added an acceleration factor to account for the phenomenon of void coalescence. This resulted in a revised description of the plastic flow of the material in the initial stage of ductile fracture. Expanding on the ideas presented in the Gurson and GNT models, there have been many studies on the analysis of the growth and coalescence of voids during ductile fracture [71,99,100,101,102,103,104,105,106].

With analytical models for describing the process of void coalescence, it is worth mentioning Brown–Embury [107] and Thomason [71,106,108] as base models. These are models developed at the micromechanical scale. The Brown–Embury model refers to a perfectly ductile material and assumes the presence of shear bands at 45° between voids. The model relates the possibility of void coalescence depending on the diameter of the voids R and the distances between their centres (X). The criterion assumes that for a given void form factor, there is a minimum relative distance between voids, below which coalescence cannot be initiated, regardless of the stress state. Thomason’s void coalescence model was developed for elastic–perfectly plastic materials, using solutions for slip lines. For the axisymmetric problem, Thomason’s model assumed that the average normal stresses affect the void if the stresses reach a certain specified value.

The aforementioned classical models relating to the growth process and the coalescence of voids assume a number of simplifications. They do not address all aspects of strain and ductile failure of real materials. Thus, there is a major role for developing in situ studies of the development of voids in materials, with a particular focus on metals. 

### 4.3. Experimental Verification of the Growth and Coalescence Process of Voids

**Figure 12:**
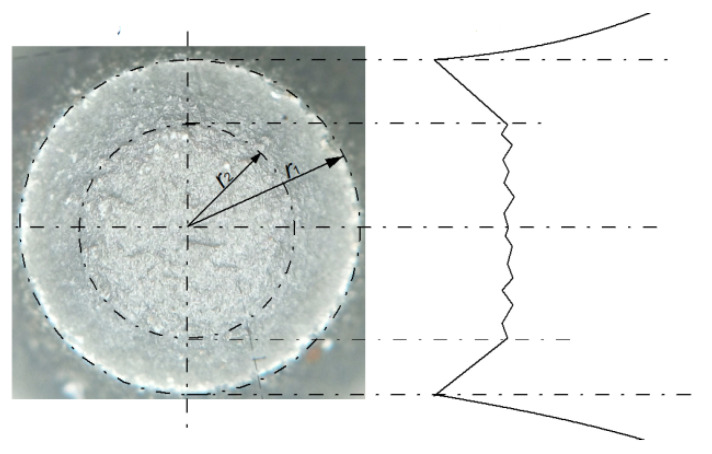
A cup-and-cone failure on a cylindrical, tensile specimen: view of break surface with scheme of the break plane profile (for S355 steel) [110].

The much more complex actual mechanism of ductile failure was highlighted in [109], taking into account the development of critical strain levels and stress triaxiality. In addition to the processes of void initiation, growth and coalescence (for tension dominated loading) and the occurrence of shear and void coalescence (for shear dominated loading), the authors point to seven types of micromechanisms that occur sequentially or are complementary in the failure process. An example of this complex type of ductile failure can be observed in the occurrence of a cup-and-cone failure on a tensile specimen, particularly for structural steels with medium-strength characteristics and high levels of ductility (Figure 12).

**Figure 13:**
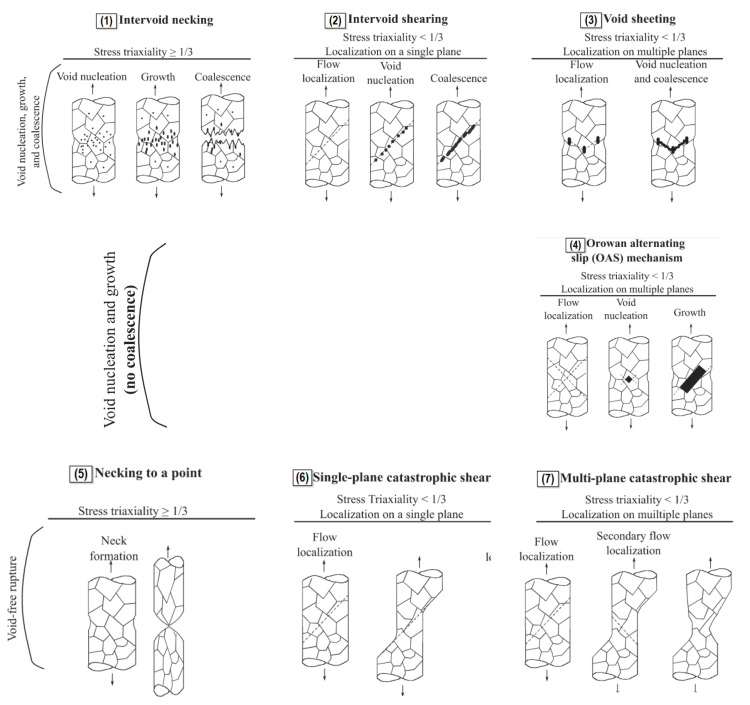
Types of ductile failure mechanisms: (**1**) Intervoid necking, (**2**) Intervoid shearing, (**3**) Void sheeting, (**4**) The Orowan alternating slip, (**5**) Necking to a point, (**6**) Single-plane catastrophic shear, (**7**) Multiplane catastrophic shear [109].

Based on an analysis of the results of experimental uniaxial tensile tests on materials such as aluminium, nickel and copper, seven different types of ductile failure mechanisms were demonstrated (Figure 13) [109]:

(1) Intervoid necking (occurring with initiation, growth and coalescence of voids): for triaxiality stress factor T ≥ 0.33.
(2) Intervoid shearing, for which the initiation and subsequent elongation of voids along shear bands is characteristic. The consequence is the coalescence of voids and the formation of macrocracks in the planes of the shear bands. The mechanism occurs for a triaxiality stress factor T less than 0.33, located in a single plane.
(3) Void sheeting, when shear develops between existing voids in the material and the simultaneous process of nucleation and coalescence of new voids. The final ductile fracture that develops connects the existing voids in the material. The mechanism occurs on multiple planes, for stress triaxiality less than 0.33.
(4) The Orowan alternating slip (*OAS*) mechanism, which assumes the occurrence of void nucleation at the intersection of slip bands and the consequent growth of prismatic voids in the results of alternating slip along shear bands. *OAS* is characterised by its occurrence on multiple planes, for a triaxiality factor T less than 0.33.
(5) Destruction by specimen necking (T ≥ 0.33).
(6) Shear leading to the destruction of a specimen, occurring in a single plane. It is realised by the sliding of the material along a single slip band. Consequently, it will lead to a loss of cohesion and failure of the specimen (triaxiality factor T ≤ 0.33).
(7) Multiplanar shear occurring along multiple shear bands; also referred to as the slipping-off mechanism (T ≤ 0.33).

The nature of the interaction in the failure process between these seven mechanisms depends on, among other things: the local state of stress, the type of microstructure of the material under consideration (microstructural changes that occur during deformation can eliminate or create void nucleation sites in the material) and the strain-hardening capacity of the material (a low strain-hardening capacity favours the interlaminar shear mechanism, while a high one predisposes to the occurrence of void sheeting). For engineering materials in the occurrence of ductile failure, the first three mechanisms identified by Noell et al. [109] will be most relevant. 

**Figure 14:**
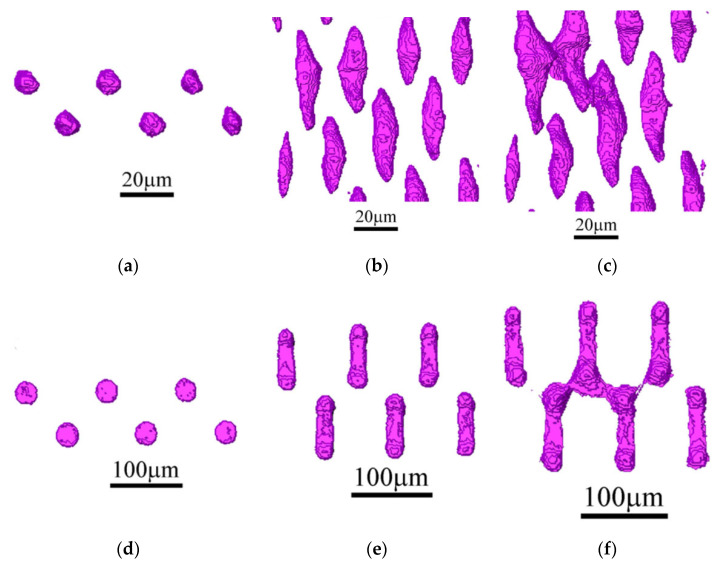
Growth and coalescence of modelled holes in copper specimens at true strain levels: (**a**) 0.00; (**b**) 0.77; (**c**) 1.01; in Glidcop alloy specimens at true strain levels: (**d**) 0.00; (**e**) 0.45; (**f**) 0.50 [80].

There are many analytical models in the literature describing ductile failure according to the void mechanism. An important aspect is the possibility of their experimental validation. Particularly difficult to validate experimentally is the phenomenon of void coalescence [111,112,113,114]. The use of X-ray computed tomography is helpful during in situ testing. In order to experimentally verify the growth process and the coalescence of voids, X-ray tomography was used for the uniaxial tensile testing of copper and Glidcop alloy in [80]. For this purpose, specimens were specially prepared for testing by laser-drilling holes in the material. Figure 14 shows the resulting images of growth and coalescence of the modelled voids. In the case of copper, the process of void coalescence occurred for true strain levels of more than 100%, while for the Glidcop alloy the strain was in the range of 50%. No secondary nucleation of voids was observed for copper. The occurrence of ripple marks on the surface of the voids was identified with the realisation of slip in the specimen. With the Glidcop alloy, coalescence was realised by nucleation of secondary voids on alumina particles between the modelled holes. Based on the results obtained, an attempt was made to verify the selected models of growth and coalescence of voids. Those models that provide quantitative verification of the results were considered. Good agreement was shown for the void growth model proposed by Rice and Tracey when taking into account the change in stress triaxiality in the specimen with progressive strain. When verifying the void coalescence process, greater discrepancies from experimental results were shown for the Brown and Embury model (differences of about 50%) than for the Thomason model (differences of 2–40%). With discrepancies in the Brown and Embury model, constraints that cause delayed void coalescence may have an impact. In the case of Glidcop alloy specimens, Thomason’s model overestimates the level of critical strain at failure due to the failure to account for the secondary void nucleation occurring in the material. 

The authors of [77] proposed a criterion for the occurrence of void coalescence in material. It is based on a modification of the assumptions of the classical Thomason criterion (and its extension by Benzerga [81]). The criteria mentioned concern the analysis of the action of normal stresses, excluding the contribution of shear components. However, shear stresses can play an important role in the process of void coalescence and subsequent crack initiation in a material. An RVE model was used, which (unlike previous models) is affected by shear components. A positive calibration of the proposed criterion for void coalescence with the results of numerical calculations for the three-dimensional model was carried out. The calibration considered the values of void form factor and void distribution. The classical Thomason criterion (with a modification by Benzerga) was generalised to any loading condition. Modifications of Thomason’s criterion for the void coalescence process, based on—among other things—the results of numerical calculations, are included in [82].

**Figure 15:**
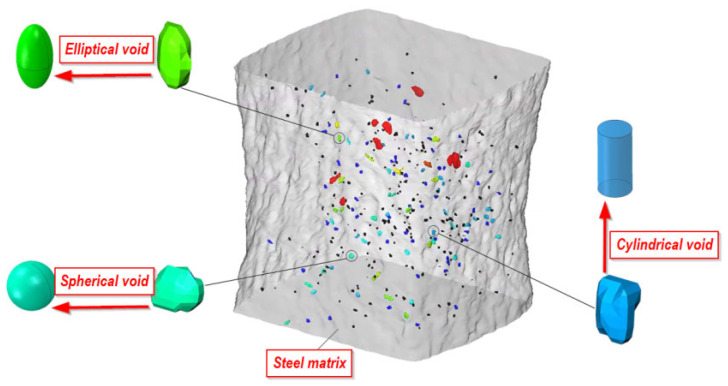
Shapes of voids in the material determined using computed tomography techniques (for structural steel) [124].

When analysing the growth process and the coalescence of voids in the material, an important aspect is to determine the volume contribution of the voids and their initial shape [115,116,117]. This allowed, among other things, for the subsequent modelling of the correct shape in the numerical model. In a number of papers, authors based on the Rice–Tracey model and the assumption of a spherically shaped void obtained oversimplified results. With the development of computed tomography methods, especially high-resolution μXCT, it has been possible to determine the true shapes of voids present in the material at the microscale level and to include them in the model describing the growth and coalescence of voids [118,119,120,121,122,123]. An example of an analysis image using the μXCT technique with the determined shape of the voids and their representation in the numerical calculation programme is shown in Figure 15 [124]. In this paper, on the basis of μXCT observations and numerical calculations, an attempt was made to determine the influence of the initial void shape (spherical, cylindrical, elliptical) on the macroscopic description of the void growth process in the material as an important stage of material destruction according to the ductile mechanism [125]. The effects of stress triaxiality, shape factor and the orientation and initial volume fraction of voids were taken into account. The influence of the initial void shape on the growth process is important at low levels of stress triaxiality. An increase in stress triaxiality reduces the difference in volume growth of voids with different shapes. For stress triaxiality above 2, almost the same increase in voids of different shapes was recorded. At low triaxiality (0.33), the volume contribution of the spherical void increases the most. The initial orientation of the voids has a strong influence on the growth process. If there is an orientation of the voids with an area located normal to the direction of stretching, greater void growth was observed. The initial volume proportion of voids showed less influence on the nature of their growth compared to the other parameters analysed [124].

The influence of the shape of the void on the nature of its growth can be determined using other advanced research methods. In [126], a research methodology was used to complement the discrete dislocation plasticity (DDP) method with calculations using XFEM [127,128]. Higher stress levels, strain hardening and void growth rates occurred under biaxial loading (compared to uniaxial loading). With a constant initial proportion of void volume, it was observed that elliptical-shaped voids showed larger surface areas relative to cylindrical voids. The voids with a larger surface area in relation to volume showed a tendency to grow faster, but with a lower proportion of strain hardening. 

**Figure 16:**
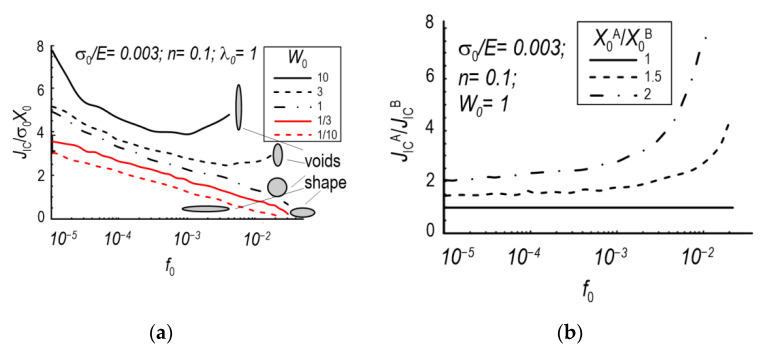
Dependence of fracture toughness level on initial porosity: (**a**) Depending on the initial shape of the void; (**b**) Depending on the anisotropic void spacing [130].

The Rice–Tracey void growth model with Huang [97] corrections was verified on the basis of CT (compact tension) analysis. Specimens from three steels (single-phase ferritic steel, two-phase steel and steel with martensitic microstructure) subjected to tension were analysed. The analyses showed an increase in voids from a few μm to 30 μm. Attention was drawn to the change in the initially spherical shapes of the voids and to the need to account for the effect of the change in stress triaxiality in the Rice–Tracey model, depending on the microstructure and characteristics of the material under study [129]. Relationships of the initial shape of voids and the distance between voids to the level of fracture toughness were developed [130], which are shown in Figure 16.

Researchers are also using advanced computed tomography techniques to verify growth models and in situ void fusion: SRCT and SRCL [131]. The idea of measurements according to these techniques is explained in [132,133]. The use of the SRCT technique carries limitations, primarily with regard to the specimens used. Specimens must have a cross-section size of approximately 1 mm for a resolution of the order of micrometres. This has consequences in terms of the use of nonstandard specimens and the influence of specimen geometry on the results obtained (influence of the presence of a plane stress region and the development of a plastic zone). In such cases, it becomes helpful to use the SRCL technique for in situ studies. This allows for a high-quality 3D image to be obtained from specimens with larger dimensions than SRCT and the inclusion of areas on the sides of the specimens. Using the SRCL technique combined with numerical calculations of the CT (compact tension) specimen [131], parameters were determined in the GNT model relating to the realisation of failure by void mechanism in the AA6061 aluminium alloy. Accurate observation of the nucleation, growth and fusion of voids is possible through the use of TEM (transmission electronic microscope) observations in experimental studies. Compared to the SEM, the TEM technique allows for the dynamic growth of voids in the material to be captured and for the void bonding process in particular to be observed at the submicron scale level. In [134], the TEM technique was used to analyse the growth and bonding of voids in different types of materials: copper and aluminium-copper alloy. 

### 4.4. Consideration of the Stress State in the Realisation of the Ductile Failure Process 

Analysis using numerical calculations (e.g., finite element method) helps understand the growth process and the coalescence of voids in detail. Very often, an elementary cell model (representative RVE volume model) is used, which in its structure contains a void or particle with specific material characteristics. Classical studies consider the analysis of cylindrical voids, spherical voids and spherical particles. Parameters that can be taken into account in the RVE loading process are the stress triaxiality parameter (T), the Lode parameter (L) and the shear coefficient (S). There has been much work involving studies of the effect of stress triaxiality on the development on ductile failure, including a description of the void growth and coalescence process. However, it has become an important task to determine the simultaneous influence of the three aforementioned parameters of stress triaxiality, Lode parameter and shear rate when analysed using RVE. A solution to the task posed was proposed in [77]. The RVE model was used in [135] to extend Gurson’s proposal to high porosity materials. The variables in the model were the volume proportion of voids and the value of the stress triaxiality factor. It was shown that the distribution of plastic deformation depends on the volume fraction of voids; a concentration of deformation occurs for a fraction with a large volume of voids. A small distance between individual voids leads to a faster coalescence process and fracture.

**Figure 17:**
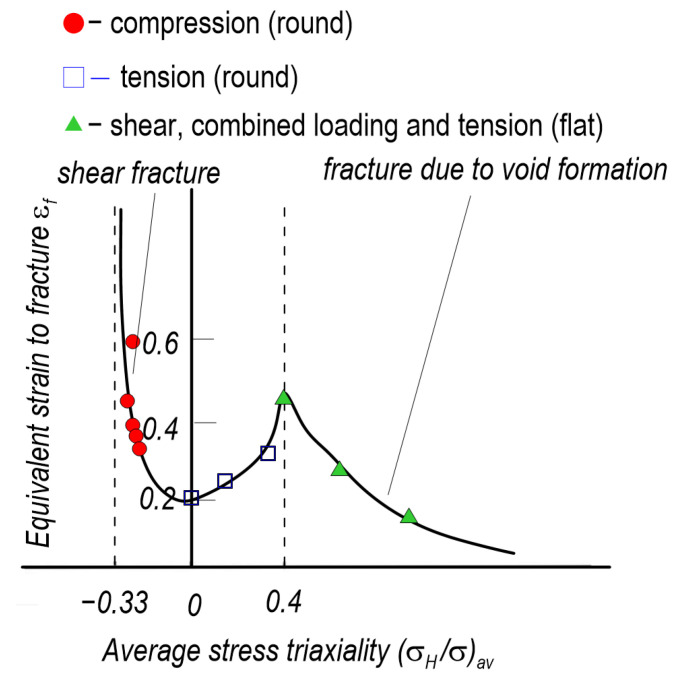
Numerically and experimentally determined dependence between critical plastic strain and the level of stress triaxiality factor (for 2024-T351 aluminium alloy) [139].

The contribution of the critical level of plastic strain, the stress triaxiality factor and the Lode parameter to the realisation of the ductile fracture process has been highlighted in a number of works by Wierzbicki and coworkers [136,137,138]. On the basis of numerous experimental studies and numerical calculations, the dependence of the critical strain at ductile fracture on the stress triaxiality was determined (Figure 17). Compression, tension and shear specimens of aluminium alloy 2024-T351 characterised by various levels of stress triaxiality factor from −0.33 to 1 were analysed. Based on the analyses, the authors concluded that shear failure occurs for compression specimens with negative values of stress triaxiality factor. For notched tensile specimens, the observed failure character was dependent on the level of stress triaxiality. For high triaxiality there was a failure mechanism involving initiation, growth and coalescence of voids. For low levels of stress triaxiality, failure was a combination between two mechanisms: void and shear. Work by Wierzbicki and colleagues and other researchers has highlighted the need to calibrate the material relationship used in the numerical calculation programme, particularly when analysing high-plasticity materials.

The influence of the Lode parameter value on the void growth and coalescence process has formed the basis of a number of papers [92,137,140,141,142,143,144]. Barsoum and Faleskog proposed a micromechanical model based on experimental studies and numerical calculations of a three-dimensional elementary cell containing a single spherical void [145]. The macroscopic stress state was defined by two variable quantities: the stress triaxiality factor and the Lode parameter. On the basis of the results obtained, it was determined that the effect of the Lode parameter on the change in the shape of voids and the rate of their growth increases as the level of the stress triaxiality factor decreases. For a stress triaxiality factor of T = 1, there is an increase and a change in the shape of the void due to shear strain. The void undergoes a change in shape from spherical to ellipsoidal, up to the onset of plastic localisation, so as to further obtain a ‘penny’ shape. For triaxiality stress level 2, there is an almost spherical increase in voids. The influence of the triaxiality parameter and Lode on the formation of plastic localisation was also determined. High values of the T parameter were accompanied by low levels of critical strain. The minimum value of critical strain occurred at a triaxiality factor of 0, while the maximum value occurred at T = 1. This marks the shear state as the most critical state from the point of view of destruction, where material failure will occur at an angle close to 45° to the plane of occurrence of the lowest principal stress [145].

## 5. Summary and Conclusions

Due to its great practical importance, the phenomenon of ductile failure associated with the development of voids in metals has been the subject of interest of researchers since the turn of the 1950s and 1960s. Over about 6 decades, many research techniques have been developed that have contributed to a better understanding of the nature of this phenomenon.

It was indicated that void formation in metals is induced by material discontinuities on the nano- and microscopic scale. The void initiators mainly include (i) intersection of the slip bands, (ii) grain boundaries, (iii) twin boundaries, (iv) vacancy clusters and (v) second-phase particles (inclusions and precipitates).

The void formation mechanism depends primarily on the material purity. In the absence of second-phase particles, voids are formed mainly in the vicinity of defects of the crystal structure (point, linear and planar defects). Materials that are commonly used in engineering (mainly steels and aluminium alloys) most often show the presence of inclusions, which, as stress concentrators, are void initiators. Depending on the geometrical and strength parameters of particles and matrix, voids are formed by particles’ separation or fracture.

As has been demonstrated, the local values of the accompanying stresses, measured on a microscopic scale, are much higher than those measured macroscopically, and range from several hundred to several thousand MPa.

After the nucleation process, voids grow with increasing strain and change their shape and position in the material. The moment of localisation of strain in the immediate vicinity of voids is identified with the initiation of the coalescence process. Three ways of realising the coalescence of voids in the material are characterised: internal necking, shear coalescence and necklace coalescence. Selected analytical models relating to the growth process and the coalescence of voids under ductile failure mechanism are cited in this paper. An important element is the possibility of experimental verification of the classical models. This verification is possible by using advanced measurement methods (computed tomography, microstructure analysis, etc.) for in situ testing. 

Despite enormous progress in this subject, there are still many unresolved issues. The course and intensity of the void development are significantly influenced by the load conditions, most importantly the stress state. However, the literature rarely mentions other factors that undoubtedly determine the development of voids, e.g., dynamic phenomena, temperature, etc.

It has not been unequivocally determined whether the stress or strain criteria must be satisfied for the void nucleation. Moreover, the literature lacks unambiguous sets of parameters describing the development of voids in materials with practical engineering application. Little is known yet about the development of voids under low-triaxiality conditions.

It should be strongly emphasised here that the observations of the development of voids are not only of cognitive importance, but above all they become the basis for the formulation of theoretical models, which further allows for the description of defective materials. It is an issue of great practical importance, thanks to which it becomes possible to perform an engineering safety assessment of structural elements containing defects, operating in pre-failure conditions. In the future, an even more accurate understanding of the ductile failure process according to the void formation mechanism will be possible with the development of advanced testing and measurement methods. These can be used for in situ research. An important aspect of supplementing the information obtained about the void mechanism will be the inclusion of the results of advanced numerical calculations.

## Data Availability

No new data were created or analysed in this study.
